# Evaluating tacrolimus treatment in idiopathic membranous nephropathy in a cohort of 408 patients

**DOI:** 10.1186/s12882-016-0427-z

**Published:** 2017-01-05

**Authors:** Hua-Zhang Qin, Lei Liu, Shao-Shan Liang, Jing-Song Shi, Chun-Xia Zheng, Qing Hou, Ying-Hui Lu, Wei-Bo Le

**Affiliations:** National Clinical Research Center of Kidney Diseases, Jinling Hospital, Nanjing University School of Medicine, Nanjing, 210002 China

**Keywords:** Membranous nephropathy, Immune suppression, Clinical nephrology

## Abstract

**Background:**

The KDIGO Clinical Practice Guidelines for Glomerulonephritis recommended tacrolimus as an alternative regimen for the initial therapy for Idiopathic membranous nephropathy (IMN), however, large observational studies evaluating tacrolimus treatment in IMN remains rare.

**Methods:**

A total of 408 consecutive IMN patients with nephrotic syndrome who were treated with tacrolimus in Jinling Hospital were included. The effectiveness and safety of tacrolimus treatment in IMN were analyzed in this study.

**Results:**

The cumulative partial or complete remission after tacrolimus therapy were 50%, 63% and 67% at 6, 12 and 24 months, respectively, and the cumulative complete remission rates were 4%, 13% and 23%, respectively. Multivariate logistic analysis showed that higher tacrolimus exposure during induction treatment, female gender, higher eGFR and no history of previous immunosuppressive therapy were independently associated with higher probability of remission. A relapse occurred in 101 of the 271 (37.3%) patients with partial or complete remission, and 18 of the 95 (18.9%) patients with complete remission. Tapering duration of tacrolimus and complete remission versus partial remission status were independent factors associated with risk of relapse. A decline in eGFR was the most frequent adverse event during tacrolimus treatment. During tacrolimus treatment, a ≥40% decrease in eGFR was observed in 43 (10.5%) patients.

**Conclusions:**

Low dose tacrolimus is effective for IMN, with a total remission rate of 66% whereas with a rather high rate of relapse. However, the safety of tacrolimus treatment needs to be further validated in large randomized clinical trials.

## Background

Idiopathic membranous nephropathy (IMN) is the most common cause of nephrotic syndrome in adults. Recent data showed that the IMN is an organ specific, antibody mediated autoimmune disease, in which the main target antigen was M-type phospholipase A2 receptor located on the podocyte [1].

The KDIGO guideline has recommended the combination of cyclophosphamide and steroids in cyclical fashion as the first line treatment for IMN, which was proved to be effective in several randomized clinical trials [2, 3]. Recently, a RCT comparing chlorambucil and cyclosporine A indicated that chlorambucil might induce a better outcome in IMN patients with deteriorated renal function at baseline [4]. However, the severe side effects induced by cytotoxic regimens like cyclophosphamide or chlorambucil restricted its wide application. Besides these regimens, rituximab, a monoclonal antibody against B cell surface antigen CD20, were reported to be able to reduce proteinuria while stabilizing renal function [5]. Although seemingly promising, large RCT study on rituximab was rare and the drug was rather expensive.

Calcineurin inhibitors (CNIs) was recommended by the KDIGO guideline as the only alternative regimens for initial therapy. Various studies has demonstrated the efficacy of CNIs (tacrolimus or cyclosporine A) in IMN [6–15]. The most limitation of these studies were the small number of the enrolled patients, and lack of large observational studies evaluating the effectiveness, safety and chance of relapse. Here, we present real-world clinical practice data from the largest IMN cohort till now of 408 patients with nephrotic syndrome from the Nanjing Glomerulonephritis Registry who were treated with tacrolimus. The aim of the study was to evaluate the effectiveness and safety of tacrolimus in the treatment of IMN.

## Methods

### Patients

A total of 706 consecutive biopsy-proven IMN cases treated with tacrolimus during follow-up in the Nanjing Glomerulonephritis Registry from February 2004 to November 2013 were screened in this study. All patients were from Chinese population. Patients who did not have NS, defined as a proteinuria ≥3.5 g/day along with serum albumin <30 g/L at the onset of tacrolimus treatment, were excluded. Patients who were lost to follow up after tacrolimus treatment (follow-up <2 months for preliminary screening), who did not follow the doctor’s orders, who exhibited coexisting glomerulonephritis and who were also treated with other immunosuppressive drugs during tacrolimus treatment were also excluded. All of these patients were clinically ruled out for secondary membranous nephropathy, including anti-nuclear antibody, Hepatitis B virus infection, blood tumor markers and abdominal ultrasound.

The pre-dose trough concentration (C0) was routinely monitored during treatment. Tacrolimus was administered at 0.03–0.06 mg/kg/d initially and adjusted to a pre-dose trough concentration level (C0) of 4 to 8 ng/mL for at least 6 months, which was then reduced gradually. Tacrolimus was given twice per day in patients included. The tacrolimus blood concentration were detected routinely at two weeks. If the target level of tacrolimus concentration was not achieved, the dosage will be adjusted and the concentration will be detected at the next follow-up, determined by clinical physicians. During the maintenance period of treatment, the concentration would only be detected when necessary (judged by clinical physicians). The reduction of tacrolimus dosage was judged by clinical physicians according to the response of treatment and patients’ tolerance. Steroid was given as 0.5 mg/kg/d as inducing treatment for two months. After two months, the steroid dose was reduced gradually (at a speed of 5 mg/month) to 10 mg and maintained for at least two years. All follow-up data were updated to July, 2015. The study was approved by Jinling Hospital Ethics Committee. Due to the retrospective nature of the study, written informed consent for participation was waived. The study adhered to the STROBE guidelines.

### Definitions

Partial remission (PR) was defined based on a urinary protein excretion >0.3 g/d, but <3.5 g/d, and a 50% or greater reduction from baseline value; confirmed by two values at least 1 week apart, accompanied by an improvement or normalization of the serum albumin concentration. Complete remission (CR) was defined by a urinary protein excretion <0.3 g/d, confirmed by two values at least 1 week apart, accompanied by a normal serum albumin concentration (>35 g/L). A relapse was defined by the reappearance of proteinuria >3.5 g/24 h after PR or CR during follow-up.

The induction period of tacrolimus treatment was defined as the first 6 months of tacrolimus therapy. For each patient, the tacrolimus exposure during inducing period (Tacrolimus exposure) was defined as the ratio of the area under the curve of blood concentration to the duration of inducing period (six months). Tacrolimus exposure = ∑_n = 1_^n^(C_n_ * T_n_)/∑_n = 1_^n^(T_n_), where n is the number of times of the blood concentration tested; C_n_ is the tacrolimus blood concentration; T_n_ is the duration of that blood concentration sustained for unchanged dosage of tacrolimus.

The CKD-EPI formula was used to estimate glomerular filtration rate (eGFR). Renal function decline was defined as a >30%, > 40% or >50% decline of eGFR when compared with baseline. A recovery of renal function deterioration was defined as the eGFR reverse to less than 15% decline compared with baseline.

### Statistical analysis

Normally distributed variables are expressed as the mean and standard deviation, and differences among groups were analyzed by t test or one-way ANOVA analysis. Qualitative data are described as percentages and analyzed using Chi-square or Fisher’s exact test. Nonparametric variables are expressed as median with interquartile range, and compared using either Mann-Whitney or Kruskal-Wallis test. Univariate followed by multiple logistic regression was used to determine associations between the clinical factors and remission of nephrotic syndrome as well. Only variables that with P value <0.1 in univariate logistic regression were further considered in the multiple logistic regression models. The cumulative remission rates of proteinuria were calculated using the Kaplan-Meier method, in which patients with no remission of proteinuria and discontinued tacrolimus treatment were considered as no remission lasting to the end of this study. The *P* value reported was two sided and *P* < 0.05 was considered statistically significant. All analyses were performed using R software (Version 3.1.2).

## Results

### Patients and baseline characteristics at the onset of tacrolimus therapy

**Fig. 1:**
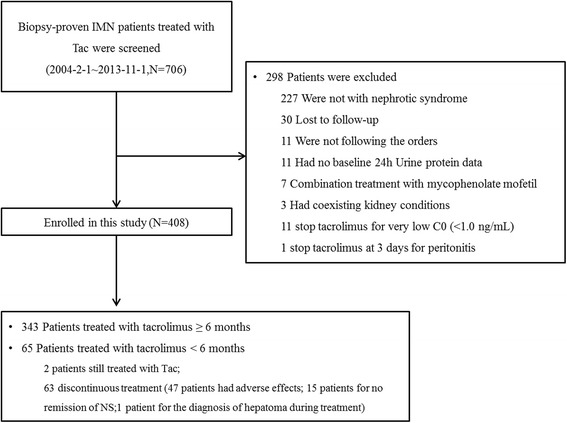
Study Enrollment. Of the 706 patients who were screened for participation in the study, 408 patients were enrolled in this study

The screening profile of the current study is shown in Fig. 1. Between 2004 and 2013, a total of 706 patients, who presented with IMN and were treated with tacrolimus during follow-up, underwent screening in this study. Of these patients, 408 were enrolled in the study. The following were reasons for exclusion: not meeting the criteria for nephrotic syndrome (defined as serum albumin < 30 g/L & proteinuria ≥3.5 g/24 h) at the onset of tacrolimus therapy (227 patients), lost to follow-up (30 patients), not following doctors’ orders (11 patients), having no baseline data regarding 24 h urinary protein or serum albumin (11 patients), combination treatment with mycophenolate mofetil (7 patients), discontinued tacrolimus treatment within two months due to substantially low tacrolimus blood concentration (<1.0 ng/ml, 11 patients), coexisting kidney conditions at baseline (a total of 3 patients: two patients with anti-GBM glomerulonephritis and one with ANCA associated glomerulonephritis), and combined with peritonitis that was not associated with tacrolimus after three days of tacrolimus treatment (one patient).

Of the 408 enrolled patients, 343 completed at least six months of tacrolimus treatment and two patients were less than six months and still under the treatment. Sixty-three patients discontinued tacrolimus treatment within six months. The main reasons for discontinuation were adverse effects (47 patients, 75%) and premature termination of tacrolimus for no remission of proteinuria (15 patients, 23.8%); in addition, one patient discontinued the tacrolimus treatment due to a diagnosis of a hepatoma during treatment. In the 408 patients, the tacrolimus treatment had been stopped by the time of last follow up in 297 patients, while continued in 111 patients. The median follow up duration after tacrolimus treatment start for patients included was 29.6 months (interquartile range, 19.3–42.9 months) and the median duration for tacrolimus treatment was 14.7 (8.3–23.3) months. Among the 297 patients who stopped tacrolimus treatment during the follow-up, 63 (21%) of them stopped within 6 months as described above, 59 (20%) between 6 and 12 months, 126 (42.4%) between 12 and 24 months, and 49 (16.4%) after 24 months.

Baseline clinical characteristics of the 408 patients are listed in Table 1. All patients showed nephrotic syndrome at the onset of tacrolimus therapy. Fifteen patients (3.7%) had an eGFR < 60 mL/min/1.73 m^2^ at baseline, but only one patient had an eGFR <30 mL/min/1.73 m^2^. A total of 71 patients (17.4%) had received previous immunosuppressive treatments, including CNIs (Eleven patients: five for tacrolimus and six for cyclosporine), corticosteroids, cyclophosphamide, and mycophenolate mofetil. During the tacrolimus treatment, 93.9% patients received concomitant treatment with steroid, and 69.4% patients with angiotensin converting enzyme inhibitors and angiotensin II receptor blockers (RASi).

**Table 1:** Characteristics of patients enrolled at baseline of tacrolimus treatment

Items	Total (*N* = 408)
Age	39 ± 16
Male gender (N, %)	298 (73%)
eGFR ^a^ at baseline	105 ± 25
eGFR < 60	15 (3.7%)
Proteinuria (g/24 h)	6.2 (4.7–8.0)
Serum albumin (g/L)	24.6 ± 3.4
Months since renal disease onset	6.6 (3.1–13.1)
Months since renal biopsy	4.6 (0.1–6.2)
Average tacrolimus doses during induction treatment	0.047 mg/kg
Average tacrolimus exposure (ng/ml) ^b^	4.7 ± 2.1
Combined with RASi§ treatment	283 (69.4%)
Combined with corticosteroids	383 (93.9%)
Previous treatment with immunosuppressive drugs	71 (17.4%)

^a^eGFR, estimate glomerular filtration rate (mL/min/1.73 m^2^), calculated by CKD-EPI formula; ^b^Six patients were not monitored blood level of tacrolimus during induction treatment; §RASi, angiotensin converting enzyme inhibitors and angiotensin II receptor blockers

### Remission of proteinuria and factors associated with the remission

**Fig. 2:**
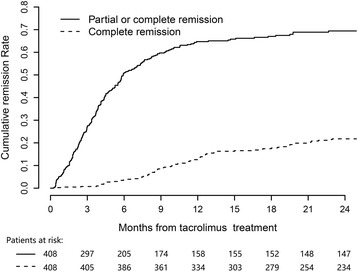
Cumulative remission rate of proteinuria during tacrolimus treatment. Patients with no remission of proteinuria and discontinued tacrolimus treatment were considered as no remission lasting to the end of this study

Of the 408 patients enrolled, 271 (66.4%) achieved partial or complete remission, and 95 (23.3%) achieved complete remission during the tacrolimus treatment. As shown in Fig. 2, the cumulative remission rates of nephrotic syndrome (partial or complete remission) after the beginning of the tacrolimus therapy, were as follows: 27% (95%CI, 22–31%), 50% (95%CI, 45–55%), 58% (95%CI, 53–63%), 63% (95%CI, 58–67%), 65% (95%CI, 60–70%) and 67% (95%CI, 61–70%) at 3, 6, 9, 12, 18 and 24 months, respectively. The cumulative complete remission rates after the beginning of tacrolimus therapy were 0.7% (95%CI, 0–2%), 4% (95%CI, 2–6%), 9% (95%CI, 6–12%), 13% (95%CI, 10–16%), 19% (95%CI, 15–22%) and 23% (95%CI, 19–27%) at 3, 6, 9, 12, 18 and 24 months, respectively. The median time to partial remission was 3.7 (2.1–6.0) months, and that to complete remission was 11.2 (8.0–17.4) months.

The clinical characteristics of patients with various responses to tacrolimus treatment (partial, complete and no remission) are shown in Table 2. There was a lower proportion of females and a shorter interval between tacrolimus treatment and the onset of renal disease, but a higher proportion of previous immunosuppressive drugs in the no remission group than the other two groups. Age, proteinuria, and serum albumin at baseline were similar among the three groups. The percentage of patients receiving corticosteroids or RASi concomitantly with tacrolimus treatment did not differ among the three groups of patients.

**Table 2:** Comparison of clinical features in patients with complete, partial, and no remission after tacrolimus treatment

Items	No Remission *N* = 137	Partial remission *N* = 176	Complete remission *N* = 95	*P-value*
Age	40 ± 16	38 ± 16	37 ± 15	0.31
Gender (Female %)	21.2%	21.6%	45.3%	<0.001
eGFR (mL/min/1.73 m^2^)	100 ± 28	107 ± 23	111 ± 22	0.008
eGFR < 60 mL/min/1.73 m^2^	6.6%	2.3%	2.1%	0.11
Proteinuria, g/24 h	6.4 (4.7–8.4)	6.1(4.8–7.9)	5.9 (4.7–7.4)	0.37
Serum albumin (g/L)	24.2 ± 3.4	24.7 ± 3.3	24.8 ± 3.4	0.30
Previous treatment with immunosuppressive drugs	26.3%	14.7	9.5%	0.002
Combined with RASi treatment	68.6%	71.5%	66.3%	0.65
Combined with corticosteroids	90.5%	94.9%	96.8%	0.11
Tacrolimus treatment since onset of disease (months)	7.6 (3.9–14.8)	7.3 (2.9–13.3)	5.1 (2.1–9.5)	0.001
Tacrolimus exposure (ng/mL)	4.2 ± 2.0	4.7 ± 2.1	5.2 ± 2.2	0.002

The induction period of tacrolimus treatment was defined as the first 6 months of therapy. For each patient, time average of blood tacrolimus exposure (tacrolimus exposure) during the induction period was defined as the ratio of the area under the curve of T0 blood concentration to the duration of the induction of the tacrolimus therapy (the first six months of tacrolimus therapy). Compared with the other two groups, patients in the no remission group had a significantly lower average tacrolimus exposure.

Factors associated with remission of proteinuria were further analyzed in logistic models (Table 3). In univariate and multivariate logistic analyses, tacrolimus exposure, gender, previous treatment with immunosuppressive therapy and eGFR at baseline were significantly associated with both partial remission and complete remission during tacrolimus treatment, whereas age and proteinuria were not. The multivariate logistic model showed that tacrolimus exposure (OR 1.2, 95% CI 1.1–1.3), gender (females vs male, OR 1.7, 95% CI 1.0–3.0), previous immunosuppressive therapy (OR 0.39, 95% CI 0.22–0.67) and eGFR at baseline (for every 10 mL/min/1.73 m^2^, OR 1.2, 95% CI 1.1–1.4) were independently associated with partial remission after tacrolimus treatment. These four factors were also independently associated with complete remission after tacrolimus treatment.

**Table 3:** Factors predicting remission of nephrotic syndrome after tacrolimus treatment in univariate and multivariate logistic model

Variables	Partial or complete remission			Complete remission		
	OR	95% CI	*P-Value*	OR	95% CI	*P-Value*
Univariate logistic models						
Gender (Female vs. Male)	1.6	0.99–2.6	0.06	3.1	1.9–4.9	<0.001
eGFR at baseline (for every 10 mL/min/1.73 m^2^)	1.1	1.0–1.2	0.003	1.1	1.0–1.2	0.06
Proteinuria at the onset of tacrolimus therapy	0.94	0.87–1.0	0.11	0.94	0.86–1.0	0.22
Previous treated with immunosuppressive drugs (Yes vs. No)	0.42	0.25–0.70	<0.001	0.42	0.19–0.85	0.02
Average tacrolimus exposure (ng/mL)	1.2	1.1–1.3	0.004	1.2	1.0–1.3	0.005
Multivariate logistic model						
Gender (Female vs. Male)	1.7	1.0–3.0	0.04	3.3	2.0–5.6	<0.001
Previous treatment with immunosuppressive drugs (Yes vs. No)	0.39	0.22–0.67	<0.001	0.40	0.17–0.83	0.02
eGFR at baseline (for every 10 mL/min/1.73 m^2^)	1.2	1.1–1.4	0.001	1.1	0.99–1.2	0.07
Average tacrolimus exposure (ng/mL)	1.2	1.1–1.3	0.001	1.2	1.0–1.2	<0.001

### Relapse of proteinuria and factors associated with the relapse

**Fig. 3:**
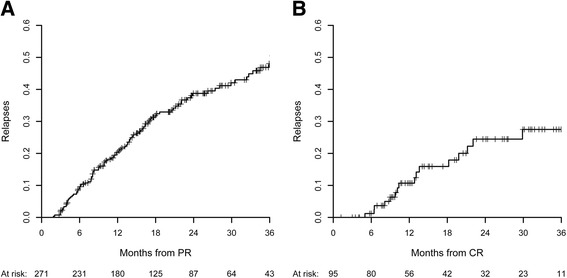
The cumulative incidences of relapse after partial remissions (3**a**) and complete remissions (3**b**)

The relapses were analyzed in 271 patients who achieved partial or complete remission during the tacrolimus treatment. A relapse occurred in 101 of those 271 patients, and 18 of whom relapsed after a complete remission of proteinuria. The median time to relapse after partial remission or complete remission was 11.5 [6–18] months, and after complete remission, it was 13 (9.2–20.8) months. As shown in Fig. 3, the cumulative incidences of relapse after partial remission or complete remission was first achieved were 10% (95% CI, 6–13%), 21% (95% CI, 16–26%), 39% (95% CI, 32–45%) and 48% (95% CI, 40–55%), respectively, at 6, 12, 24 and 36 months, whereas the cumulative incidences of relapse after complete remission was first achieved were 1% (95% CI, 0–3%), 11% (95% CI, 3–17%), 24% (95% CI, 12–35%) and 27% (95% CI, 14–39%), respectively, at 6, 12, 24 and 36 months.

It’s notable that 65.3% of relapse occurred before tacrolimus treatment stopped. At relapse, 36 patients had stoped tacrolimus treatment, while 65 patients maintained at an average dosage of 2.3 mg/d. Among 101 patients who relapsed during follow-up, 34 patients didn’t receive the steroid treatment at the time of relapse, while 67 patients remained the steroid treatment at an average dosage of 11.3 mg/d (one patients 2.5 mg/d, forty-eight patients between 5 mg-10 mg/d, and eighteen patients > 10 mg/d). We also analyzed factors associated with relapse of proteinuria in 175 patients who achieved partial remission after the treatment and discontinued tacrolimus during follow up. In a multivariable COX analysis adjusting for age, gender, baseline eGFR, the CR/PR status (HR = 0.2, 95% CI 0.1–0.4, *P* < 0.001) and longer tapering duration (HR 0.97, 95%CI 0.94–0.99, *P* = 0.006) were independent protective factors for the relapse. The longer tapering duration, the lower risk of relapse.

### Adverse events

**Fig. 4:**
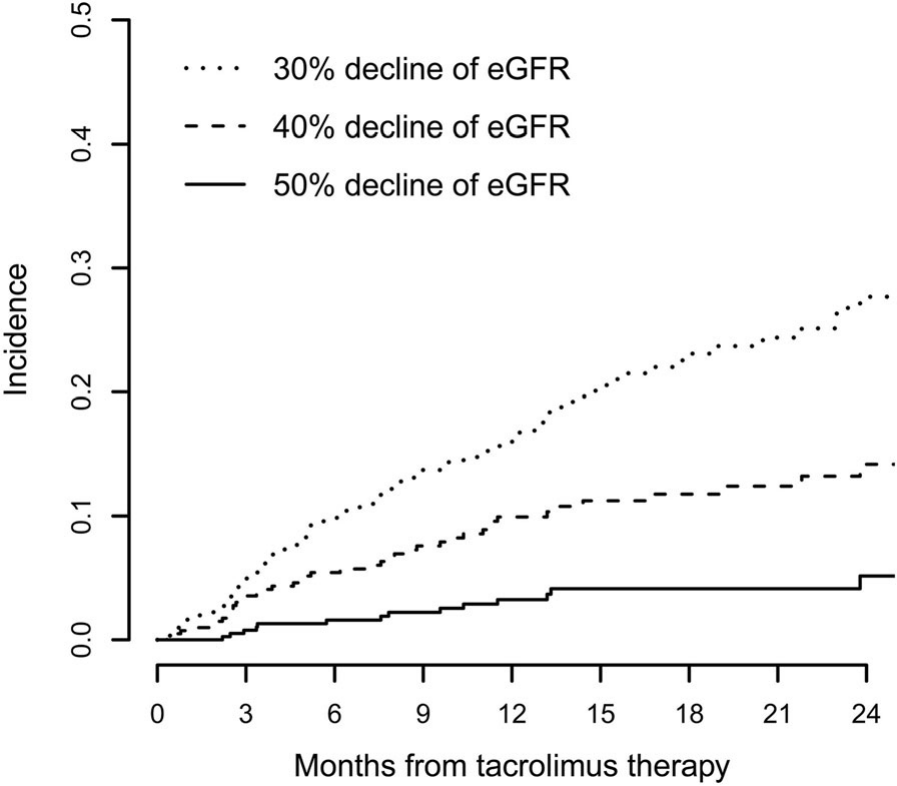
The incidences of decrease of eGFR during the treatment of tacrolimus

A decline in eGFR was the most frequent adverse event during tacrolimus treatment. A decline in eGFR may be caused by nephrotoxicity of tacrolimus or progression of nephrotic syndrome, which is difficult to differentiate in clinical practice. However, once the decline in eGFR occurred during tacrolimus treatment, nephrotoxicity of tacrolimus should be considered for safety concerns. The cumulative incidence of decline in eGFR is shown in Fig. 4. The development of decline in eGFR is time dependent, and it can occur at any time during tacrolimus treatment. The cumulative incidences of a ≥30% decrease in eGFR during tacrolimus treatment were 9% (95% CI, 7% to 13%), 16% (95% CI, 12% to 20%) and 28% (95% CI, 22% to 33%) at 6, 12 and 24 months, respectively; the cumulative incidences of a ≥40% decrease in eGFR were 6% (95% CI, 3% to 7%), 10% (95% CI, 7% to 13%) and 15% (95%CI, 10% to 20%) at 6, 12 and 24 months, respectively. The cumulative incidences of a ≥50% decrease in eGFR were 2% (95% CI, 0% to 3%), 3% (95% CI, 1% to 5%) and 6% (95% CI, 2% to 10%) at 6, 12 and 24 months, respectively.

**Fig. 5:**
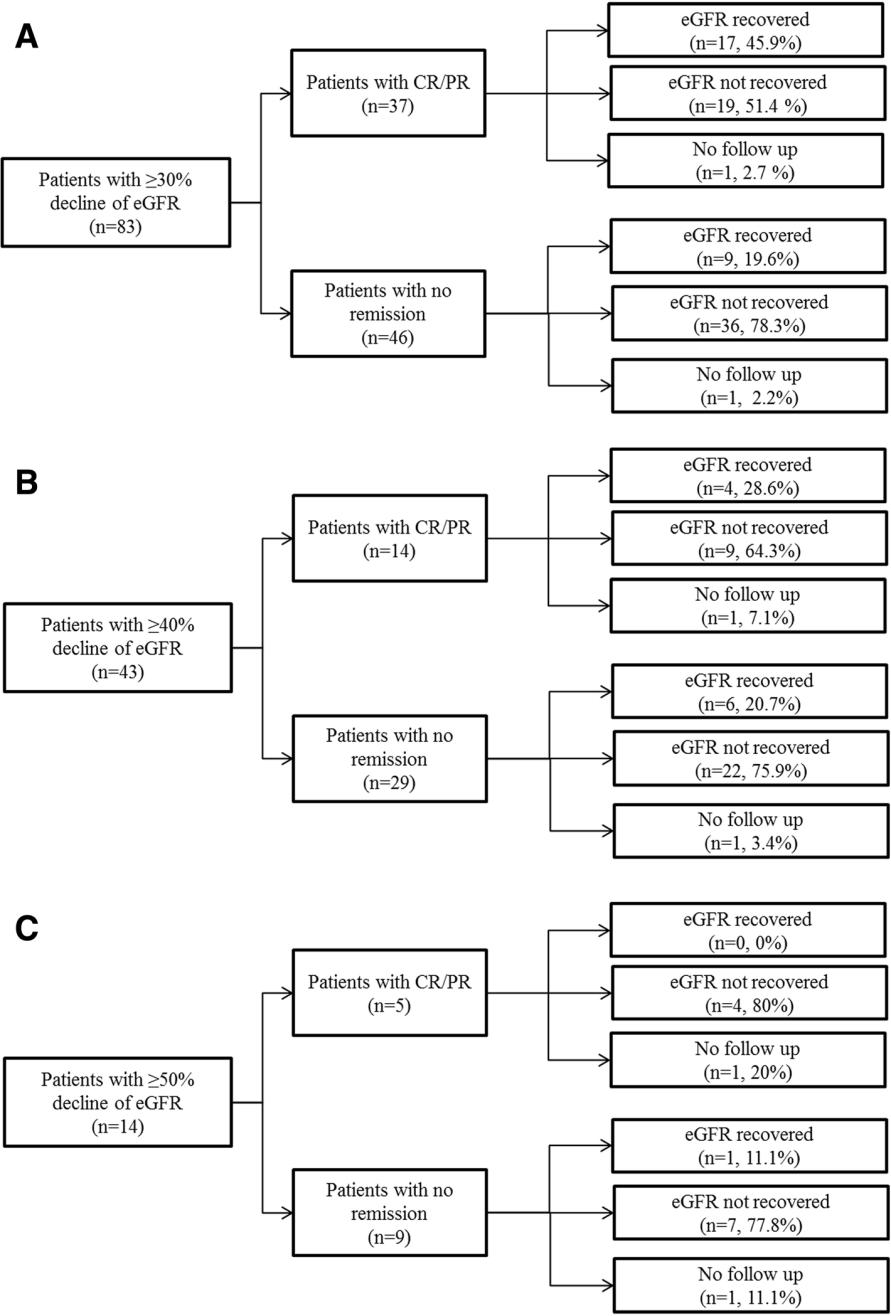
Follow up of patients with ≥30 (**a**), 40 (**b**) or 50% (**c**) eGFR decrease. A recovery of renal function deterioration was defined as the eGFR reverse to less than 15% decline compared with baseline

Of the 83 patients with ≥ 30% decline of eGFR, the tacrolimus was discontinued in 57% patients, reduced in 30% patients and sustained in 13% patients. CR/PR status was achieved before eGFR event occurred in 37 patients, seventeen (45.9%) of these patients had eGFR recovered in the follow up. Forty-six patients had sustained proteinuria when eGFR event occurred, nine (19.6%) of them had eGFR recovered in the follow up (Fig. 5a). Of the 43 patients with a ≥ 40% decrease in eGFR. Tacrolimus treatment was discontinued or tapered in 40 (93%) patients. Fourteen of these patients showed a partial or complete remission of nephrotic syndrome before the eGFR event occurred, in which 4 patients (28.6%) had their eGFR recovered in the follow up. Twenty-nine patients had sustained proteinuria and six of these patients (20.7%) had eGFR level recovered (Fig. 5b). Of the 14 patients with a ≥ 50% decrease in eGFR, tacrolimus treatment were all discontinued or tapered. There were 5 patients achieved partial or complete remission of nephrotic syndrome before eGFR event and none of them had eGFR recovered in the follow up. Nine patients had no remission of nephrotic syndrome and only one of them had eGFR level recovered (11.1%) (Fig. 5c).

Other notable adverse reactions are shown in Table 4. Infections occurred in 36 cases (8.8%, 14 cases were treated in the hospital), newly developed hyperglycemia occurred in 28 cases (6.9%), neurological adverse reactions developed in 31 cases (7.6%) and cardiovascular adverse reactions (Six patients had tachycardia; one patient had bradycardia; one patient had tachycardia and angina; one patients had angina) occurred in 9 patients (2.2%).

**Table 4:** Other adverse events associated with tacrolimus treatment

Adverse events	Number of patients
Total Infections	36 (8.8%)
Respiratory system	13
Skin	8
Digestive system	9
Urinary system	6
Nervous system	1
Hospital treated infections	14
Hyperglycemia	28 (6.9%)
Neurological	31 (7.6%)
Tremor	16
Blurred vision	12
Muscle trembling or twitching	6
Agitation	2
Mental depression	1
Headache	1
Cardiovascular system	9 (2.2%)

## Discussion

Our study shows that a remission of nephrotic syndrome was observed in 66% of the examined Chinese patients with IMN during tacrolimus treatment. The cumulative remission rates (partial or complete remission) was 50% and 63% within 6 and 12 months, respectively, after the onset of tacrolimus therapy in the current study (Fig. 2). The remission rate of tacrolimus therapy in this study is slightly lower than that in previous studies [7–9, 15]. A small RCT showed that the remission rate of proteinuria was 72% (18/25) after 12 months of tacrolimus monotherapy compared with 22% (5/23) in the supportive therapy group [9]. Another small RCT from China showed that the remission rate (partial or complete remission) was significantly higher in the tacrolimus group (85%, 33/39) than in the CTX group after 6 months of treatment (65%, 22/34) [8]. Recently, a study in India had observed remission rate of 71% at 12 month with tacrolimus treatment [15]. The lower remission rate observed in the current study than in previous studies can be explained by the finding that the tacrolimus blood levels in this study were relatively lower than those in previous studies [8, 16]. It’s notable that the tacrolimus treatment was continued in 111 patients at the end of follow up, the remission rate may go higher if we keep following up of these patients. Furthermore, tacrolimus treatment was discontinued in 63 patients for various reasons within six months of the treatment, and most of those patients were considered to have no remission during the tacrolimus treatment. If those patients who discontinued tacrolimus treatment before remission were considered censored observations, the Kaplan–Meier method would dramatically over estimate the remission rate.

Tacrolimus exhibit highly individual variations in pharmacokinetics and pharmacodynamics Therefore, therapeutic drug monitoring of tacrolimus is universally applied in solid organ transplantation and treatment of glomerular disease. We found that tacrolimus exposure during the treatment induction period is independently predictive of the probability of remission in this study: the higher the tacrolimus exposure, the higher the probability of remission. However, under higher exposure, risk of side effect, especially nephrotoxicity also went higher, which made the decision of tacrolimus doses difficult.

One of the most concern of tacrolimus treatment was its nephrotoxicity. In this study, renal function decline (defined by >40% decline in eGFR) was seen in a total of 43 patients (10.5%). Previous Studies showed that renal function decline was largely observed in patients received tacrolimus for immunosuppression after kidney or non-kidney transplantation and other autoimmune disease [17–21]. Higher tacrolimus exposure, though resulted in a higher remission rate, may at the same time increase the incidence of renal function decline. Tacrolimus related nephrotoxicity is associated with both irreversible or reversible histologic damage to all compartments of the kidneys, including the glomeruli, arterioles, and tubulo-interstitium, but the non-specificity of most lesions makes the differential diagnosis regarding other injurious processes cumbersome [19]. However, it’s worth to mention that in some patients the renal function decline might be attributed to the development of IMN but not tacrolimus. In clinical practice, we could hardly differentiate tacrolimus related nephrotoxicity or renal damage from sustained proteinuria related renal function decline. We also lacked a biomarker that could specifically predict tacrolimus related nephrotoxicity. In our study, there were four patients who achieved CR/PR before ≥40% eGFR decline occurred and had their eGFR level recovered after tacrolimus treatment tapered or withdrawal. The renal function decline in these four patients should be attributed to tacrolimus related nephrotoxity. There were also nine patients who achieved CR/PR and with irreversible eGFR decline after treatment withdrawal. In these patients, tacrolimus related nephrotoxicity should also be carefully considered. It’s worth to mention that in patients with a remission but renal function decline > 50%, none had their eGFR recovered, indicating that tacrolimus might have resulted in an irreversible renal function damage in these patients. Taken together, our data indicated that tacrolimus treatment might related to considerable renal function decline while lack specific biomarker for identifying nephrotoxity.

In addition to tacrolimus exposure, female gender, no history of previous immunosuppressive therapy and a higher eGFR at baseline were independently associated with a higher probability of remission during tacrolimus therapy. The associations between these three factors and remission were also observed in a recent study [16]. However, the amount of proteinuria at baseline was not significantly associated with the probability of remission.

A high rate of relapse was also observed in this study. Patients with partial remission were followed by a higher likelihood of relapse than those with complete remission. A previous study suggested that the relapses may be partly prevented by a longer tapering period [16]. In the current study, we also found the longer tapering duration, the less chance of relapse. However, longer tacrolimus treatment could also associated with an increased incidence of nephrotoxicity. The optimal tapering strategy remains to be determined.

There exist some limitations in the current study. Due to its retrospective nature, it was rather hard to control the time duration of tacrolimus treatment, which may influence the efficacy observed. It’s also important to realize that in fact IMN patients can achieve spontaneous remission rate under only supportive treatment (ACEIs or ARBs) [15, 22], so the remission rate observed in the current study might over estimate tacrolimus efficacy to some extent. To precisely describe the efficacy of tacrolimus, a well-controlled clinical trial shall be carried out in the future.

## Conclusion

Low dose tacrolimus is effective for IMN. Renal function decline was not rare in patients treated with tacrolimus. Besides the safety concerns, a rather high relapse rate was also of great consideration in tacrolimus treatment. Our findings validates the effectiveness of tacrolimus in a large number of IMN patients, however, large RCT studies still seems to be necessary before more extensive use of tacrolimus.

## Abbreviations

ACEIs: Angiotensin converting enzyme inhibitors
ARBs: Angiotensin II receptor blockers
CI: Confidence interval
CKD-EPI: Chronic kidney disease epidemiology collaboration
CNIs: Calcineurin inhibitors
CR: Complete remission
CTX: Cyclophosphamide
eGFR: Estimate glomerular filtration rate
HR: Hazard ratio
IMN: Idiopathic membranous nephropathy
KDIGO: Kidney disease, improving global outcomes
OR: Odds ratio
PR: Partial remission
RASis: Ras inhibitors
RCT: Randomized clinical trial
